# Comparison of three genomic DNA extraction methods to obtain high DNA quality from maize

**DOI:** 10.1186/s13007-016-0152-4

**Published:** 2017-01-03

**Authors:** Amani Abdel-Latif, Gamal Osman

**Affiliations:** 1 Department of Botany and Microbiology, Faculty of Science, Alexandria University, Alexandria, Egypt; 2 Department of Biology, Faculty of Applied Sciences, Umm Al-Qura University, PO Box 715, Makkah, 21955 Kingdom of Saudi Arabia; 3 Agricultural Genetic Engineering Research Institute (AGERI), Giza, 12619 Egypt

**Keywords:** Internal transcribed spacer (ITS), Internal transcribed spacer 2 (ITS2), DNA extraction

## Abstract

**Background:**

The world’s top three cereals, based on their monetary value, are rice, wheat, and corn. In cereal crops, DNA extraction is difficult owing to rigid non-cellulose components in the cell wall of leaves and high starch and protein content in grains. The advanced techniques in molecular biology require pure and quick extraction of DNA. The majority of existing DNA extraction methods rely on long incubation and multiple precipitations or commercially available kits to produce contaminant-free high molecular weight DNA.

**Results:**

In this study, we compared three different methods used for the isolation of high-quality genomic DNA from the grains of cereal crop, *Zea mays*, with minor modifications. The DNA from the grains of two maize hybrids, M10 and M321, was extracted using extraction methods DNeasy Qiagen Plant Mini Kit, CTAB-method (with/without 1% PVP) and modified Mericon extraction. Genes coding for 45S ribosomal RNA are organized in tandem arrays of up to several thousand copies and contain codes for 18S, 5.8S and 26S rRNA units separated by internal transcribed spacers ITS1 and ITS2. While the rRNA units are evolutionary conserved, ITS regions show high level of interspecific divergence and have been used frequently in genetic diversity and phylogenetic studies. In this study, the genomic DNA was then amplified with PCR using primers specific for *ITS* gene. PCR products were then visualized on agarose gel.

**Conclusion:**

The modified Mericon extraction method was found to be the most efficient DNA extraction method, capable to provide high DNA yields with better quality, affordable cost and less time.

## Background

The extraction of good quality DNA with a high yield is a limiting factor in plants’ genetic analysis. DNA quality from each line should be consistent to allow a proper genetic analysis from several plant individuals. High quality of DNA is characterized by predominantly high molecular weight fragments with an A260/280 ratio between 1.8 and 2.0 and the lack of contaminating substances, such as polysaccharides and phenols [1]. The extraction and purification of high-quality DNA from cereals is generally difficult due to the presence of polysaccharides, proteins, and DNA polymerase inhibitors such as tannins, alkaloids, and polyphenols. The presence of these compounds effects the quality and quantity of isolated DNA, and therefore, renders the sample non-amplifiable [2]. Polysaccharides, the most commonly found contaminants in plant DNA extraction, make DNA pellets slimy and difficult to handle. The anionic contaminants inhibit restriction enzymes and effect enzymatic analysis of the DNA [3]. DNA extraction using dry seeds of wheat, barley, rice, and other cereals for RFLP and PCR based analyses of plant genotypes and genetic variation has been studied earlier [4–6]. Pure and rapid DNA extraction is a pre-requisite for most advanced techniques such as genetic mapping, fingerprinting, marker-assisted selection, and for evaluating authenticity of exported cereal varieties. The extraction of high-quality DNA from plant tissue is time consuming, arduous, and costly due to multiple steps and the high cost of liquid nitrogen. In addition, the problems associated with the available commercial kits are their high cost and low yield of DNA [5, 7]. Several methods to isolate DNA from plant tissues are available; however, these methods produce either small amounts or DNA of inconsistent quality. Most of the DNA extraction methods are modified versions of cetyltrimethyl ammonium bromide (CTAB) extraction with some crop-to-crop limitations and differ in time and cost. The main cause of the differences in the CTAB protocol is the composition of cell walls and intracellular components such as nucleus mitochondria and cellulose. CTAB is a cationic surfactant added in the DNA extraction buffer, which dissociates and selectively precipitates DNA from histone proteins [27]. The lignification of cereal cell walls makes its degradation difficult and thus limits DNA extraction. Although commercially available column-based extraction kits are effective in isolating contaminant-free DNA from recalcitrant plant species, there is still loss of significant amounts of DNA on the column. The Mericon method provides fast and easy DNA purification in convenient spin column format. Typical yields are 3–30 μg of high-quality DNA, depending on the samples used. The purification of DNA using the DNeasy Plant Mini Kit method was modified to simplify the protocol and maximize DNA yield. DNA quality required for PCR and sequencing is often very high with DNA of high molecular weight and with less shearing, free of contamination from protein, RNA or polysaccharides, and 260/280 nm absorbance ratio of approximately 1.8–2.0. A fast, simple, and reliable DNA extraction method, which does not require long incubations, multiple DNA precipitations, or commercial reagents, and could meet the PCR, sequencing, and next-generation library preparation requirements, will be invaluable to plant research. Therefore, the aim of this study was to compare quality and quantity of DNA isolated using three different extraction methods.

## Methods

### Plant material and tissue disruption

*Zea mays* grains (M10 and M231) were obtained from the Crop Institute, Agricultural Research Center, Giza, Egypt. The grains were soaked in water for 24 h at 25 °C and the embryos were isolated from them using scalpel. The grains were crushed in mortar to obtain fine powder and 100 mg of each sample was transferred to an Eppendorf tube. In parallel, 100 mg of the grain pieces were ground into a fine powder using liquid nitrogen.

### DNA extraction

#### Qiagen-method

Maize DNA was extracted using a commercially available kit (DNeasy Plant Mini Kit, Qiagen, Valencia, CA, USA) following the manufacturer’s instructions. The DNeasy membrane from QIAGEN combines the binding properties of a silica-gel based membrane with micro spin technology. DNA is adsorbed to the DNeasy membrane in the presence of high salt concentrations of chaotropic salt, which removes water from hydrated molecules in solution. In DNeasy extraction procedure, buffer conditions are designed to allow adsorption of DNA specific to the silica-gel membrane and offer an optimal removal of carbohydrates, polyphenols and other plant metabolites. The time consumed in this method was about 1.5–2 h for 10 samples.

#### CTAB-based method

The genomic DNA was extracted from 100 mg of each sample by CTAB-based method according to Inga et al. [8] with slight modification. The sample was mixed with 300 μL sterile deionized water, 500 μL of CTAB buffer (20 g CTAB/L, 2.56 M NaCl, 0.1 M Tris–HCl, 20 mM EDTA) and 20 μL proteinase K (20 mg/mL). In parallel extraction, 1% polyvinylpyrrolidone (PVP) was added. The samples were incubated for 1.5 h at 65 °C and 20 μL RNase A (10 mg/mL) was added. Then the mixture was incubated in a thermo-shaker water bath (65 °C) for 10 min. The samples were subjected to centrifugation at 16,000×*g* for 10 min and supernatants were extracted twice with 500 μL chloroform. The upper phase was transferred to a new tube and incubated at room temperature for 1 h after mixing it with double volume of CTAB precipitation solution (5 g/L CTAB, 0.04 M NaCl). The samples were centrifuged for 5 min at 16,000×*g* and supernatants were discarded. The remaining precipitates were dissolved in 350 μL of 1.2 M NaCl and 350 μL chloroform, and centrifuged at 16,000×*g* for 10 min. The upper phase was transferred to another tube, mixed with 0.6 volume of isopropanol and centrifuged at 16,000×*g* for 10 min. The supernatant was discarded and pellet was washed with 500 μL of ethanol (70% v/v). After centrifugation, the supernatant was carefully discarded, the pellet was dried for 1 h, and DNA was dissolved in 100 μL sterile deionized water. The genomic DNA was extracted following CTAB-based method used to extract DNA from seeds of soybean, wheat, barley, oats, maize, and rice [9, 10]. This method took about 3–4 h for 10 samples.

#### Modified Mericon extraction method (Qiagen DNeasy Mericon Kit)

Maize DNA was extracted using commercially available kit (Qiagen DNeasy Mericon Kit), following the manufacturer’s protocol. The sample, 100 mg (5 × 20 mg), was added to a 2-mL Eppendorf tube containing 1 mL Lysis Buffer and 2.5 µL proteinase K solution, mixed thoroughly and incubated in a thermo-shaker for 30 min at 65 °C and 1000 rpm, and allowed to cool to room temperature (20 °C). The entire content of Eppendorf tube was transferred to a pre-filter (Analytik Jena), centrifuged at 13,000×*g* for 5 min and 700 µL of supernatant was further transferred into a new 2-mL Eppendorf tube. After adding 500 µL of chloroform to the supernatant, the samples were mixed vigorously and centrifuged at 13,000×*g* for 15 min at 4 °C. The upper phase was carefully collected, mixed with 500 µL chloroform and centrifuged again at 13,000 rpm for 15 min at 4 °C. The supernatant (in 250 µL batches) was collected in a 2-mL Eppendorf tube and mixed carefully with 1 mL phosphate buffer. Then 600 µL of this solution was transferred to a QIAquick spin column and centrifuged at 17,900×*g* for 1 min and filtrate was discarded. The remaining batches of the same sample were similarly applied to respective columns and subsequent steps were followed. Then 500 µL of AW2 (70% ethanol to wash the salts out) was added to the QIAquick spin column and centrifuged at 17,900×*g* for 1 min. After discarding the filtrate, the column was again centrifuged at 14,000×*g* for 1 min. The DNA was eluted into fresh 1.5 mL Eppendorf tube by adding 30–100 µL elution buffer to QIAquick spin column and incubating it for 5 min at room temperature and then centrifuging at 14,000×*g* for 1 min. Modified Mericon extraction method took ~1 h for 10 samples.

### Spectrophotometric analyses of DNA

The concentration, purity (A260/A280 ratio), and absorbance ratio at 260–280 nm (A260/A230 ratio) were measured with a Thermo Scientific NanoDrop™ 1000 Spectrophotometer (Thermo Scientific, Germany) using 1 µL of each sample. The spectra were recorded for a range of 220–750 nm.

### PCR amplification

The internal transcribed spacer (ITS) of nuclear ribosomal DNA, one of the most commonly used DNA markers in plant phylogenetic and DNA barcoding analyses, is recommended as a core plant DNA barcode [11]. For polymerase chain reaction (PCR) analysis, each DNA sample was diluted to a working concentration of 20 ng/µL. ITS regions were amplified in a Peltier Thermal Cycler (Bio-Rad Laboratories Inc., Germany) using the universal primers. The ITS region (including ITS1, 5.8S and ITS2) of each sample was amplified with forward primers P1: 5-TCGTAACAAGGTTTCCGTAGG-3 and reverse P2: 5-TCCTCCGCTTATTGATATGC-3 [12]. The ITS region I between the 18S rDNA and the 5.8S rDNA is flanked by ITS 5 and ITS 2; and ITS region II between the 5.8S rDNA and the 28S rDNA is flanked by ITS 3 and ITS 4 and it should amplify 700 bp [12]. The oligonucleotides were synthesized and purified by MWG Biotech. PCR reaction was carried out in a final volume of 12.5 μL containing 0.5 μL of DNA-template, 12 μL of Taq master mix (Qiagen Biotech Co., Germany) supplemented with Taq DNA polymerase (Jena Bioscience, Jena, Germany). Another PCR master mix was used to amplify DNA extracted using Mericon extraction. PCR thermal cycling conditions were initial denaturation at 94 °C for 3 min, followed by 35 cycles of denaturing at 94 °C for 1 min, annealing at 61 °C for 1 min, extension at 72 °C for 1 min, with a final extension at 72 °C for 10 min [13].

### Agarose gel electrophoresis

DNA was analyzed by agarose gel electrophoresis using 1, 1.5, and 2% agarose gel (SeaKem LE agarose, Cambrex, gels for genomic and amplified DNA). Electrophoresis was performed using 1× Tris–Borate EDTA (TBE) buffer containing 1 μg/mL of ethidium bromide (EtBr) and a constant voltage of 100 V for 50 min. The DNA bands were visualized and images were acquired using Gel Doc XR+ Imaging system (Bio-Rad Laboratories Inc., Germany).

## Results and discussion

Since the first use of CTAB-based method for extraction of DNA from plant leaves [14, 15], it has been modified several times to reduce contaminants such as polyphenols and polysaccharides that are present in the plant tissues [16–18]. Although all currently published methods of DNA extraction have demonstrated their effectiveness in isolating DNA that is suitable for PCR amplification or restriction digestion, they require long incubations, multiple precipitation steps, and ethanol washes to produce RNA-free genomic DNA of high purity. These additional manipulations reduce overall yield and may fail to produce large amounts of high quality of DNA.

### DNA quality and quantity assessment

**Fig. 1:**
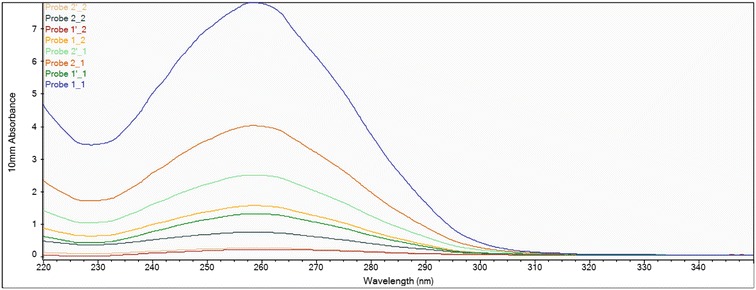
Nano-Drop measurement profile of genomic DNA extractions from *Z. mays*. DNA extractions using Mericon extraction method. Probe = Sample

**Fig. 2:**
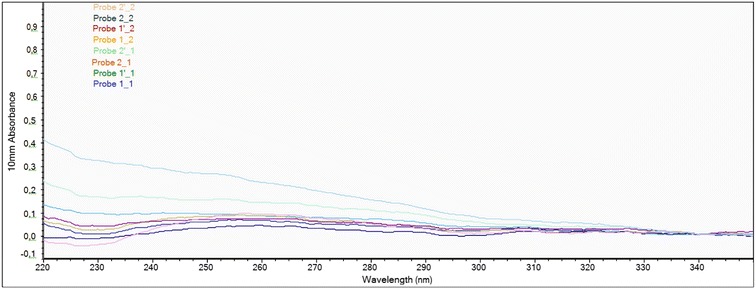
Nano-Drop measurement profile of genomic DNA extractions from *Z. mays*. DNA extractions using a CTBA-based extraction method with and without 1%PVP.Probe = Sample

**Fig. 3:**
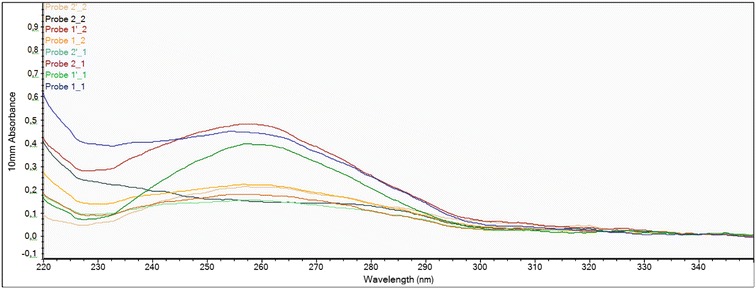
Nano-Drop measurement profile of genomic DNA extractions from *Z. mays*. DNA extractions using a Qiagen extraction method. Probe = Sample

 The quality of each extracted DNA sample was verified spectrophotometrically using a NanoDrop instrument and agarose gel electrophoresis. The NanoDrop absorbance profile is useful for detection of contaminants such as protein, salts, and polysaccharides, which can inhibit and interfere in DNA sequencing. The 260/280 nm ratio of 1.8 indicated that the extracted DNA had high purity with absence of proteins and phenols. The overall DNA yield was in a range of 100–200 ng per 100 mg of homogenized material, which is sufficient to conduct 200 PCR reactions. Table 1 summarizes the DNA yield and purity range obtained for all sample extracts using the three extraction methods. Since matrix effect was reduced by using the same samples, the variations in the data can be attributed to the effects of extraction methods. A 260/280 ratio in this study was found to be in a range of 1.2–2.07. A 260/280 ratio between 1.93 and 2.27 indicates insignificant levels of contamination [19, 20]. Most authors used liquid nitrogen or freeze-drying for primary extraction. In this study, high quality DNA was extracted without the use of liquid nitrogen. The purity of DNA varied with the method of extraction (Table 1). DNA purity can be severely affected by various components of sample matrices such as polysaccharides, lipids, and polyphenols or extraction chemicals like CTAB. The Qiagen method produced DNA samples with purity ratios in a range of 1.2–1.95 whereas the purity ratio of samples extracted by CTAB was between 1.6 and 2.0. A purity ratio of >1.9 indicates the presence of RNA in the sample. The ratio of <1.7 in few samples of DNA extracted by CATB method suggests the presence of proteins in those samples. These differences could be explained by the ability of some of the procedures in elimination of contaminating molecules. Sufficient purity does not guarantee successful amplification of a gene; there are also other factors such as concentration that also need consideration [21, 22]. High purity DNA was extracted from M 10 and M 321 using the three different extraction methods is shown in Figs. 1, 2 and 3. The properties of high purity DNA extracted using the three extraction methods are compared in Tables 2, 3 and 4. High quality DNA is characterised by 260/280 absorbance ratio of approximately 1.8 with a single absorbance peak at 260 nm. The DNA concentrations were higher among the samples obtained using modified Mericon extraction method (Fig. 1) compared with that obtained with the CTAB-based extraction (Fig. 2) or Qiagen method (Fig. 3). The ratio obtained varied from 1.6 to 1.8 indicating that the isolated DNA was free from contamination [23]. The spectrophotometric profile showed maximum absorbance ratio (260/280) of 2.7. The highest DNA yield was obtained from M10 hybrid by Mericon extraction method for the first elution (386.9 ng/µL) and second elution (63.2 ng/µL). Similarly, the DNA content of first and second elution of M 321 isolated by Mericon extraction method was 198.3 and 123 ng/µL, respectively. Concerning the comparison between the three methods in terms of saving time for 15 samples preparation: Qiagen-Method Consumed time from 1.5 to 2 h, CTAB-based method Consumed time from 3 to 4 h and Modified Mericon extraction method took ~1 h for 15 samples. Also it worth mentioned that the cost of Qiagen DNA extraction kit for 250 samples equal 1200$ (US).

**Table 1:** Summary of DNA extraction methods used in this study

DNA extraction method	Basis and format	Starting material	Extraction buffer	Elution buffer	Maximum DNA yield (ng DNA/mg sample)	DNA purity A260 nm/A280 nm ratio
Qiagen	Silica membrane binding; spin-column format	100 mg	400 µL buffer A	150 µL buffer A	23.6	1.80–1.95
CTAB	Solution-based; selective precipitation of DNA	100 mg	1000 µL buffer (2% CTAB, 1.4 M NaCl, 20 mM EDTA, 100 mM Tris HCl pH 8.0)	500 μL of ethanol solution (70%)	11.2	1.2–2.90
Mericon extraction	Silica membrane binding; spin-column format	100 mg (6 × 50 mg)	1000 µL buffer (20 mM EDTA, 100 mM Tris HCl pH 8.0)	EB: 10 mM Tris–Cl, pH 8.5	386.9	1.61–2.00

**Table 2:** DNA yield and purity range obtained for all sample extracts using the Mericon extraction method with and without using liquid nitrogen (N)in the extraction

#	Sample ID	User name	Nucleic acid conc.	Unit	A260	A280	260/280	260/230	Sample type	Factor
1	Probe 1_1	M10 1st elution	386.9	ng/µL	7.737	3.733	2.07	2.28	DNA	50.00
2	Probe 1′_1	M10 2nd elution	63.2	ng/µL	1.265	0.610	2.07	3.27	DNA	50.00
3	Probe 2_1	M321 1st elution	198.3	ng/µL	3.966	1.942	2.04	2.36	DNA	50.00
4	Probe 2′_1	M321 2nd elution	123.0	ng/µL	2.460	1.215	2.02	2.45	DNA	50.00
5	Probe 1_2	M10 1st elution, liquid N	75.9	ng/µL	1.518	0.735	2.07	2.56	DNA	50.00
6	Probe 1′_2	M10 2nd elution, liquid N	8.7	ng/µL	0.175	0.098	1.78	−8.55	DNA	50.00
7	Probe 2_2	M321 1st elution, liquid N	35.2	ng/µL	0.704	0.356	1.98	2.18	DNA	50.00
8	Probe 2′_2	M321 2nd elution, liquid N2	11.1	ng/µL	0.221	0.114	1.93	4.28	DNA	50.00

**Table 3:** DNA yield and purity range obtained for all sample extracts using the CTBA-based extraction method with and without 1%PVP

#	Sample ID	Usezr name	Nucleic acid conc.	Unit	A260	A280	260/280	260/230	Sample type	Factor
10	Probe 1_1	M10 1st elution	11.2	ng/µL	0.223	0.149	1.50	0.70	DNA	50.00
11	Probe 1′_1	M10 2nd elution	4.4	ng/µL	0.088	0.045	1.98	−1.99	DNA	50.00
12	Probe 2_1	M321 1st elution	1.9	ng/µL	0.038	0.013	2.90	−2.15	DNA	50.00
13	Probe 2′_1	M321 2nd elution	3.4	ng/µL	0.068	0.049	1.38	1.83	DNA	50.00
14	Probe 1_2	M10 1st elution, 1%PVP	6.9	ng/µL	0.138	0.106	1.31	0.85	DNA	50.00
15	Probe 1′_2	M10 2nd elution, 1%PVP	4.0	ng/µL	0.080	0.050	1.60	3.83	DNA	50.00
16	Probe 2_2	M321 1st elution 1%PVP	3.9	ng/µL	0.079	0.066	1.20	0.87	DNA	50.00
17	Probe 2′_2	M321 2nd elution 1%PVP	2.9	ng/µL	0.059	0.037	1.61	27.96	DNA	50.00

**Table 4:** DNA yield and purity range obtained for all sample extracts using the Qiagen extraction method with and without using liquid nitrogen (N) in the extraction

#	Sample ID	User name	Nucleic acid conc.	Unit	A260	A280	260/280	260/230	Sample type	Factor
1	Probe 1_1	M10 1st elution	21.8	ng/µL	0.435	0.251	1.73	1.12	DNA	50.00
2	Probe 1′_1	M10 2nd elution	19.3	ng/µL	0.386	0.201	1.93	5.62	DNA	50.00
3	Probe 2_1	M321 1st elution	8.5	ng/µL	0.171	0.102	1.67	2.08	DNA	50.00
4	Probe 2′_1	M321 2nd elution	7.3	ng/µL	0.147	0.103	1.43	1.66	DNA	50.00
5	Probe 1_2	M10 1st elution, N	10.7	ng/µL	0.213	0.135	1.58	1.61	DNA	50.00
6	Probe 1′_2	M10 2nd elution	23.6	ng/µL	0.472	0.256	1.84	1.71	DNA	50.00
7	Probe 2_2	M321 1st elution, N	7.0	ng/µL	0.140	0.123	1.14	0.62	DNA	50.00
8	Probe 2′_2	M321 2nd elution, N	10.2	ng/µL	0.203	0.134	1.51	4.06	DNA	50.00

### Visualizing DNA by agarose gel electrophoresis

**Fig. 4:**
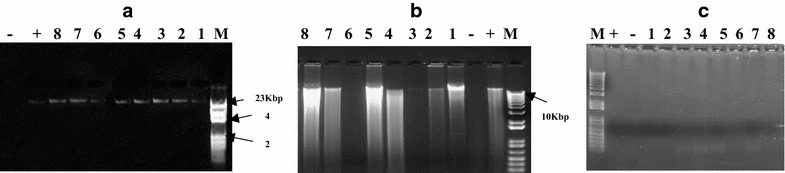
Agarose gel electrophoresis showing genomic DNA preparation of two *Z. mays* hybrids M10 (*lanes 1*–*4*) and M321 (*lanes 5*–*8*). DNA extractions using the Mericon extraction method with different agarose concentrations, 1% (**a**), 1.5% (**b**) and 2% g agarose (**c**), *lane−* empty, *lane+* positive Probe NTC. M A: λ DNA-*HindIII* marker, M B and C: one Kb Marker

**Fig. 5:**
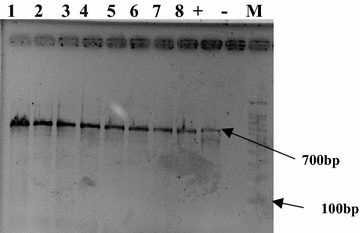
Amplified ITS of the plant materials used in the present study. M10 (*lanes 1*–*4*) and M321 (*lanes 5*–*8*). *Lane M* marker GelPilot 100 bp ladder (Qiagen)

In the present study, three different agarose concentrations (1, 1.5 and 2% agarose were used. The best results were obtained in 1% agarose gel (Fig. 4). Gel electrophoresis revealed a single, high molecular weight DNA band with little evidence of shearing and absence of RNA contamination. Figure 5 shows the results of PCR amplification of genomic DNA isolated from maize using the three different extraction methods. All extracts had positive amplification except for the CTAB extraction (Lanes 5–8 and 13–16). The DNA samples extracted were appeared as distinct bands separated on gel at their corresponding high molecular weight. None of the DNA samples showed significant smearing, which indicates degradation of sample. The present study also tried to optimize the genomic DNA extraction method by modifying CTAB protocol. NaCl in extraction buffer is responsible for the removal of proteins and carbohydrates that are attached to the DNA. The addition of high molar concentrations of NaCl to the extraction buffer (the original CTAB protocol) increases the solubility of polysaccharides in ethanol, thereby effectively inhibiting co-precipitation of the polysaccharides together with the DNA [24–26]. Muhammad et al. [27] isolated high quality and quantity of DNA from roots, leaves, and seeds using modified CTAB protocol. Among all tested NaCl concentrations (0.85, 1.39, 1.71, 2.56, and 3.42 M), the concentration 2.56 M resulted in the maximum DNA yield from any tissue source [27]. In this study, however, addition of 2.56 M NaCl to the extraction buffer used in CTAB method did not help in isolation of useable DNA from the two *Z. mays* hybrids. In addition, the maximum yield obtained from seeds was 240 ng/µL [27] while ours was 386.9 ng/µL. During incubation at 65 °C, the extraction solution became brown and became precipitated; however, no DNA was detected on agarose gel. PVP (1–2% w/v) has been successfully used in CTAB-based extractions of DNA from plant species to absorb polyphenols and prevent oxidation of polyphenols, which renders DNA unusable for downstream application [17, 28]. In this study, however, the addition of 1% PVP to the CTAB extraction method failed to isolate DNA from both *Z. mays* hybrids. Although NanoDrop measurements revealed the absorbance peak at 260 nm, 2% agarose gel electrophoresis did not show a DNA band. The PCR-amplified DNA fragments of ITS for all samples showed a clean single band product when examined on an agarose gel (Fig. 5). The PCR products were of about 700 bp.

## Conclusion

In this study, three DNA extraction methods were compared to isolate high quality DNA that can be efficiently amplified using PCR. The mechanical grinding of cells directly in the DNA isolation buffer was found to be a very simple method and more cost effective than the use of liquid nitrogen. Among the DNA extraction methods used in this study, the modified Mericon extraction method was found to be the most efficient in isolating high DNA yield with better quality from *Z. mays* hybrids. The DNA extracted using this protocol can be used for whole-genome sequencing, advanced sequencing technologies, and bioinformatic tools. Our results also indicated that maize seeds, which gave maximum DNA yield of 386.9 ng/µL, can be used as the main source of genomic DNA extraction. Interestingly, the addition of 1% PVP to the CTAB–based extraction method failed to isolate DNA from grains of two *Z. mays* hybrids.

## Abbreviation

ITS: the internal transcribed spacer
